# Genome Assembly of a Relict Arabian Species of *Daphnia* O. F. Müller (Crustacea: Cladocera) Adapted to the Desert Life

**DOI:** 10.3390/ijms24010889

**Published:** 2023-01-03

**Authors:** Waleed Hamza, Khaled M. Hazzouri, Naganeeswaran Sudalaimuthuasari, Khaled M. A. Amiri, Anna N. Neretina, Shamma E. S. Al Neyadi, Alexey A. Kotov

**Affiliations:** 1 Biology Department, College of Science, United Arab Emirates University, Al Ain P.O. Box 15551, United Arab Emirates; 2 Khalifa Center for Genetic Engineering and Biotechnology, United Arab Emirates University, Al Ain P.O. Box 15551, United Arab Emirates; 3 A.N. Severtsov Institute of Ecology and Evolution, Russian Academy of Sciences, 119071 Moscow, Russia

**Keywords:** *Daphnia arabica*, desert, genome, water flea

## Abstract

The water flea *Daphnia* O.F. Müller 1776 (Crustacea: Cladocera) is an important model of recent evolutionary biology. Here, we report a complete genome of *Daphnia (Ctenodaphnia) arabica* (Crustacea: Cladocera), recently described species endemic to deserts of the United Arab Emirates. In this study, genome analysis of *D. arabica* was carried out to investigate its genomic differences, complexity as well as its historical origins within the subgenus *Daphnia* (*Ctenodaphnia*). Hybrid genome assembly of *D. arabica* resulted in ~116 Mb of the assembled genome, with an N50 of ~1.13 Mb (BUSCO score of 99.2%). From the assembled genome, in total protein coding, 5374 tRNA and 643 rRNA genes were annotated. We found that the *D. arabica* complete genome differed from those of other *Daphnia* species deposited in the NCBI database but was close to that of *D.* cf. *similoides*. However, its divergence time estimate sets *D. arabica* in the Mesozoic, and our demographic analysis showed a great reduction in its genetic diversity compared to other *Daphnia* species. Interestingly, the population expansion in its diversity occurred during the megadrought climate around 100 Ka ago, reflecting the adaptive feature of the species to arid and drought-affected environments. Moreover, the PFAM comparative analysis highlights the presence of the important domain SOSS complex subunit C in *D. arabica*, which is missing in all other studied species of *Daphnia*. This complex consists of a few subunits (A, B, C) working together to maintain the genome stability (i.e., promoting the reparation of DNA under stress). We propose that this domain could play a role in maintaining the fitness and survival of this species in the desert environment. The present study will pave the way for future research to identify the genes that were gained or lost in this species and identify which of these were key factors to its adaptation to the harsh desert environment.

## 1. Introduction

In recent years, water fleas (Crustacea: Cladocera) have become important models for geneticists and ecologists. These organisms are commonly used in studies that test ecological and evolutionary theories due to easy culturing, short generation time, and clonal reproduction [1,2]. However, despite the long history of cladoceran investigations, many aspects of their taxonomy, evolutionary history, and even biology are not adequately known.

A genomic approach can deal with the above problems, and a species of the genus *Daphnia* O.F. Müller, 1776, was among the first organisms to be subjected to such studies. The species was “*D.* (*Daphnia*) *pulex* Leydig, 1860” [3,4], although it was another taxon with dubious status. Following this, the genomes were studied in *D.* (*D.*) *galeata* Sars, 1864 [5], *D.* (*Ctenodaphnia*) *magna* Straus, 1820 [6,7], and other species of this genus, along with other members of the family Daphniidae [8]. Genomic methods have become much more accessible over the past five years. This has allowed geneticists to expand their studies from the most studied genus of the Cladocera—*Daphnia* to other families: Bosminidae [9], Chydoridae [10], and Sididae [11,12,13]. Full-genome phylogenies of the cladocerans have been proposed recently [8,14,15]. However, we are still very far from understanding the principles of the whole genome structure in cladocerans, and the accumulation of species-specific genome data is a very important step in this work. 

We still lack adequate data on the species composition of the cladocerans inhabiting areas with extreme natural conditions such as deserts, which cover huge areas of the Earth’s surface. Cladocerans from such regions were objects of some morphological studies in the past [16,17,18,19], but up to now, biology and genomic adaptations to hard conditions of such creatures have been inadequately studied by comparison with other animals (e.g., mammals) [20].

The Arabian Peninsula is desert terrain in the Middle East. It has a vast land area covering around 2,590,000 km^2^. The Arabian Peninsula is an arid desert region that receives precipitation of less than 100 mm/year [21], while evaporation is 10 times greater than precipitation, leading freshwater scarcity [22,23,24]. The United Arab Emirates (UAE), in particular, has no permanent streams or regularly accumulating bodies of surface freshwater. Flash flooding is one of the characteristics of the area. This mostly occurs in the eastern UAE, and is usually accompanied by violent, short-lived rainstorms. The flash floods surge from the mountain toward the proximal ends of the watersheds, along valleys, and thence toward the Gulf of Oman in the east, or toward the desert in the west. A few previous studies of cladocerans have been conducted in this region using morphological identification [25,26,27]. 

Recently, we established a program of cladoceran studies using genetic methods, in which we demonstrated the pre-Pleistocene relict status of some taxa [28] and found a very specific species of *Daphnia* (*Ctenodaphnia*) Dybowski et Grochowski, 1895, namely, *D*. (*C*.) *arabica*, known to derive from a single shallow water body that completely dries up in summer [29]. The aim of this article was to present the complete genomic analyses of this *Daphnia* species and reveal its differences from other species at the genomic level. The genomic adaptations of this species to extreme conditions will be explored.

## 2. Results

### 2.1. Genome Assembly and Characterization

**Figure 1:**
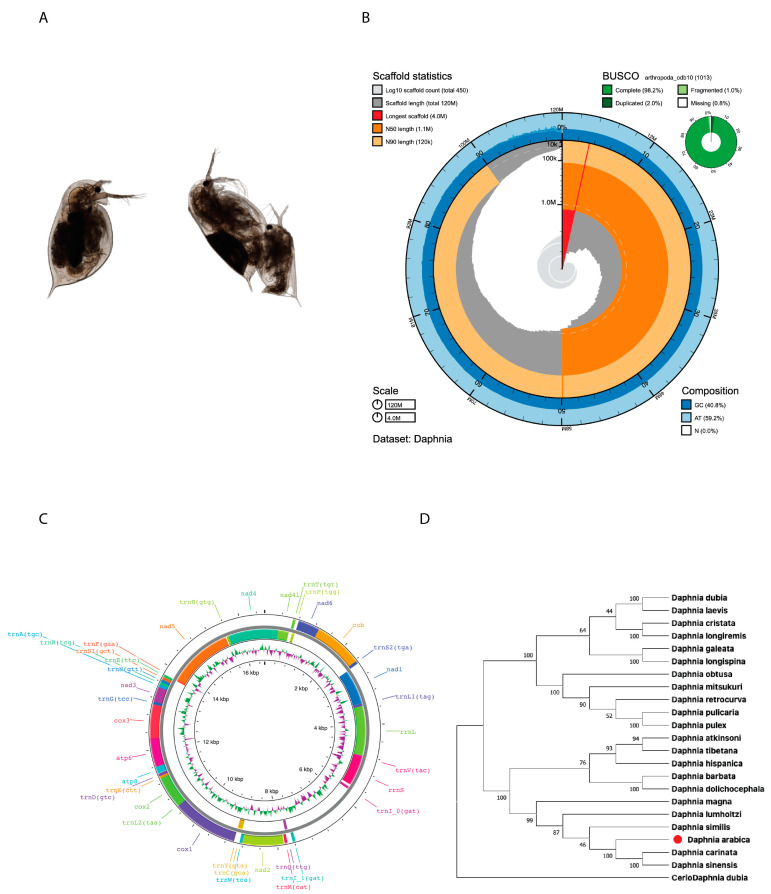
(**A**) General views of *Daphnia arabica:* parthenogenetic female and ephippial female and male at copulation. (**B**) *D. arabica* whole genome assembly statistics and assembly quality in snail plot view. (**C**) *D. arabica* mitogenome map. (**D**) Mitogenome based phylogenetic tree of *D. arabica*.

In this study, we generated 60.6 Gb (523 X) of *D. arabica* whole genome sequencing (WGS) data (Table S1) for whole genome assembly. The hybrid de novo genome assembly resulted in a draft genome with the size of ~116 Mb. The assembled genome size was more than ~18% of the theoretically estimated haploid genome size (~98 MB; without repeats) (Figure S1). In total, 454 contigs were obtained from the assembly with an N50 value of ~1.13 Mb and GC% of ~40.8 (Figure 1B). Furthermore, genome completeness was confirmed by BUSCO analysis, which found 99.2% of arthropod orthologous genes (single copy: 96.2%, duplicated: 2%, and fragmented: 1%) from the assembly (Figure 1B). The finally assembled draft genome with N50 (1.13 Mb) was comparable to the finished published genomes of other *Daphnia* species (Table S2). From the final assembly, mitochondrial genome-related contig (size 16,588 bp) was separated. The assembly statistics of both the nuclear and mitochondrial genome are given in Table 1. From the mitogenome, we annotated 13 proteins coding 23 tRNA and 2 rRNA genes (Table S3 and Figure 1C). Our original mitogenome-based phylogenetic analysis showed that the assembled *Daphnia arabica* was evolutionarily closely related to subgenus *Daphnia* (*Ctenodaphnia*): *Daphnia carinata, D. magna, D. similis,* and *D. sinensis*) (Figure 1D).

**Figure 2:**
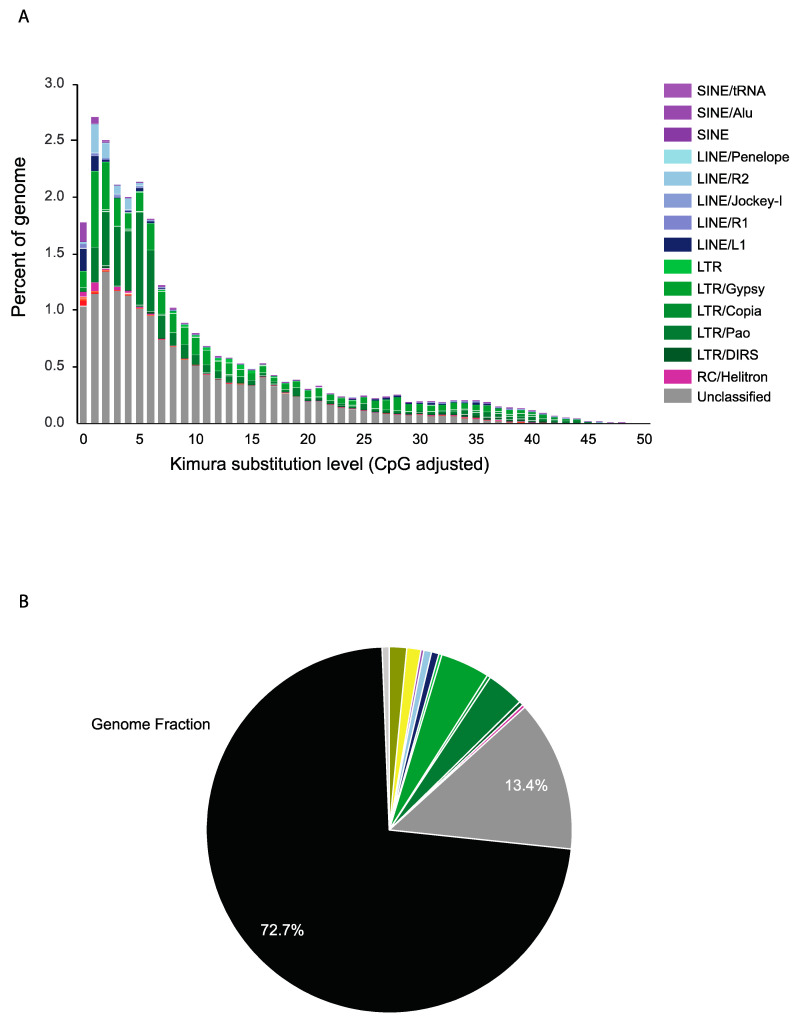
(**A**) Repeat landscape annotation in *D. arabica* genome. (**B**) *D. arabica* whole genome characterization with black color of the pie chart reflecting the total genome not occupied with repeats.

Our repeat analysis identified 13.33% of the genome repeats (Figure 2A,B). We observed an abundance of long terminal repeats (LTRs) as well as tRNA/SINEs and LINEs. From the repeat masked genome, we annotated 24,041 proteins, coding the 5374 tRNA and 643 rRNA genes (Table 1). Based on the similarity search against NCBI-NR and the Uniprot-trEMBL Protein Database using BlastP program (e-value: 0.000001), ~89% of the predicted genes were functionally annotated (Tables S4 and S5). Furthermore, we annotated 13,823 protein sequences using InterProScan and obtained protein domain-related information (Table S6). Based on KEGG pathway analysis, 6411 metabolic pathway-related proteins were identified (Table S7). Among the revealed genes, the C subunit of SOSS (sensor of ssDNA) was detected, missing in all of the other studied taxa of *Daphnia*. Many possible fragments of a viral origin were detected, previously found in other daphniids [30], but were not discussed here.

**Figure 3:**
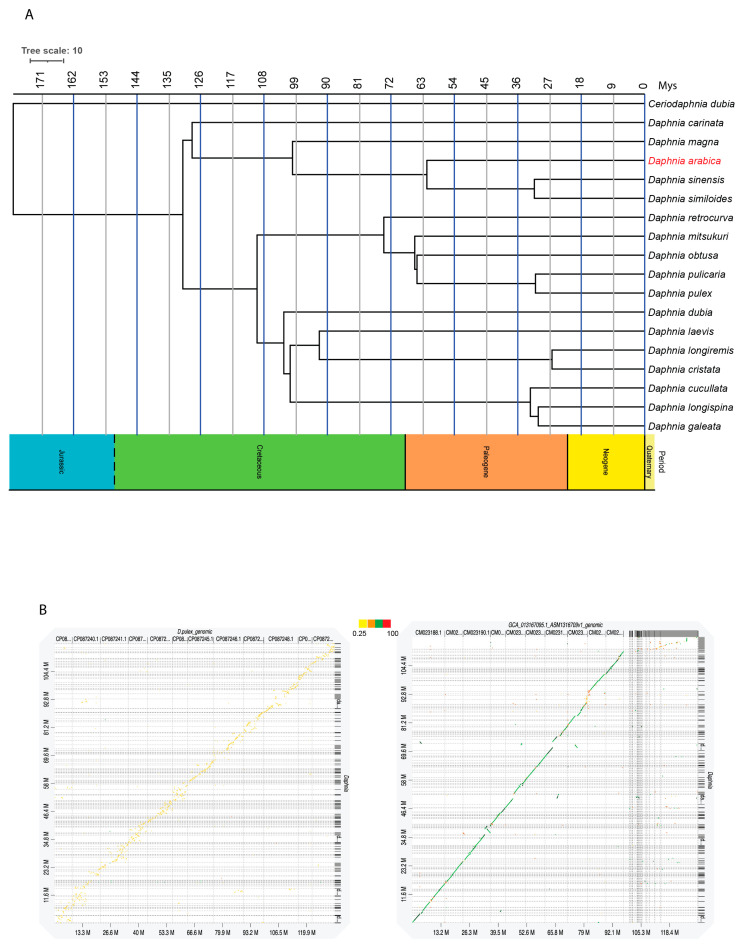
(**A**) Whole genome shot-gun sequence-based divergence time estimation of *D. arabica.* (**B**) Pairwise genome comparison *D. arabica* vs. *D. pulex* (**left**) and *D. arabica* vs. *D. sinensis* (**right**).

Based on our data, we concluded that *D. arabica* shares the highest homology with *D. sinensis,* which was expected, as the former belongs to the *D. sinensis* species complex *sensu* Hamza et al. [29]. Our whole genome synteny analysis showed the same results with more similarity to *D. sinensis* (80%), with the similarity dropping to 25% when compared to *D. pulex* (Figure 1D and Figure 3A,B). Note that the set of studied species was somewhat different in Figure 1D and Figure 3A as full genomes are known for a smaller number of taxa compared to mitogenomes.

### 2.2. Diversity and Comparative Genome Analysis

**Figure 4:**
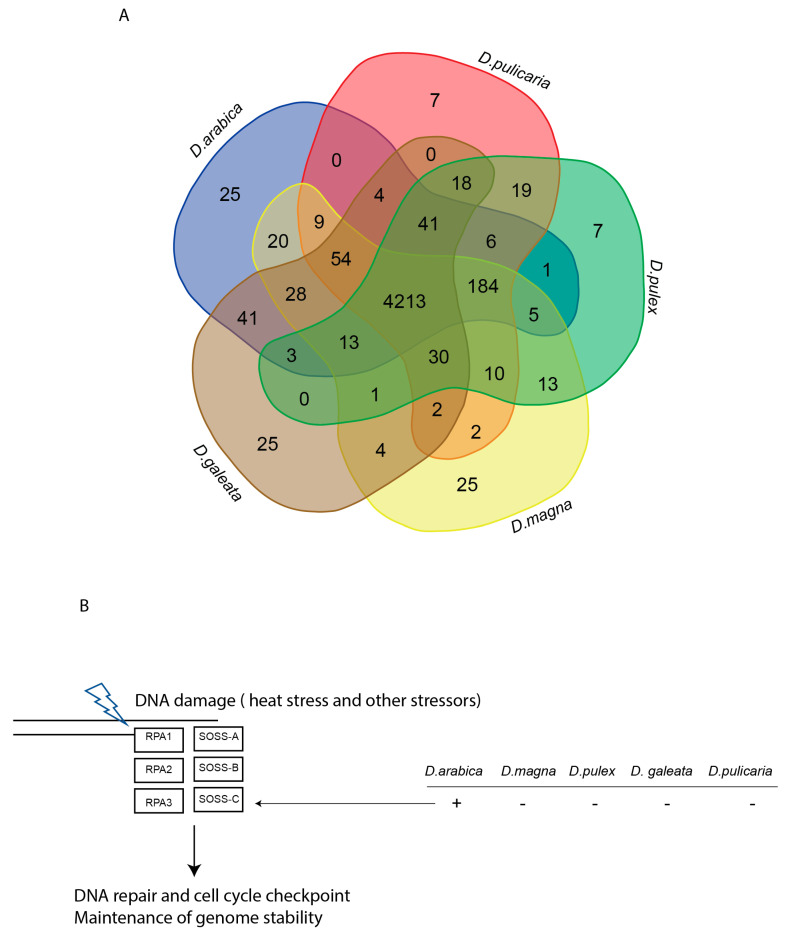
Comparative analysis based on the Daphnia gene families. (**A**) Venn diagram shows the distribution of PFam gene families between five Daphnia species (*D. arabica, D. pulicaria, D. pulex, D. magna and D. galeata).* (**B**) Comparison of SOSS-C in *D. arabica, D. pulicaria, D. pulex, D. magna,* and *D. galeata*.

Our diversity analysis results showed a reduction in diversity (Pi=) in *D. arabica* compared to other *Daphnia* species (Figure 4A). PFAM comparative genome analysis identified 4213 shared domains among the compared *Daphnia* species, and interestingly, 25 domains that are unique to *D. arabica* (Figure 4A). We conducted a manual curation to make sure that the unique identified PFAM were all real by performing a blast against the nr database. Our results showed that there was only one PFAM unique to *D. arabica* but missing in the other *Daphnia* species (Figure 4B), which belonged to the subunit C of the SOSS complex.

### 2.3. Evolution and Demographic History

**Figure 5:**
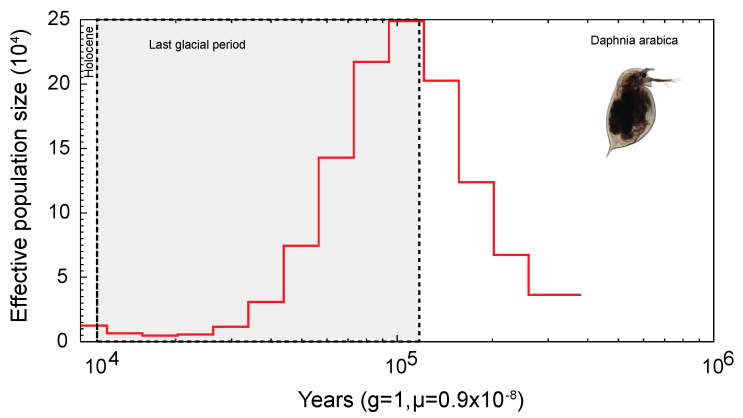
*D. arabica* effective population size reduction estimation.

Our phylogenetic results confirm that the newly isolated species of *D. (C.) arabica* is an old species that is closer to *D. (C.)* cf. *similoides* and *D. (C.) sinensis* than the other *D. (Ctenodaphnia)* species from the *D. (C.) sinensis* group sensu Hamza et al. [29] (Figure 3A). Our rough estimation of the differentiation timing led to the conclusion of a Paleogene (c.a. 60 MYA) differentiation of the *D. similis*-complex (*D. similis* + *D.* cf. *similoides* + *D. arabica*), and approximately the same differentiation time of the *D. arabica* clade. In contrast, the demographic history analysis showed a relatively “recent” bottleneck reflected by the reduction in the effective population size, then an expansion of this species took place around 100,000 years ago (Figure 5).

## 3. Discussion

In the study, we present the first complete genome of a relict microscopic crustacean, *Daphnia (Ctenodaphnia) arabica*, recently discovered in the desert of the United Arab Emirates. It is a very old lineage; a divergence time estimate using whole genome unassembled data for phylogenetic analysis dates the species divergence at the Paleogene. This is consistent with the estimations by Hamza et al. [29] based only on three mitochondrial genes. Moreover, the entire Arid Belt of Eurasia could be particularly rich in pre-Pleistocene freshwater relicts [28], but such a hypothesis needs statistically accurate confirmation based on several cladoceran and non-cladoceran taxa.

Note that the results of our previous mtDNA analysis were by chance dependent on a part of the mitochondrial genome used in that study. It has been mentioned that molecular techniques such as mtDNA sequence and barcoding have been introduced as supporting tools capable of shedding light on genetic differences between morphologically similar species [31,32]. However, molecular analyses have experienced many difficulties, especially in the consequent use of different software to analyze the resulting DNA barcodes [33]. Moreover, the mtDNA has substantial limitations, since it only describes the history of a single locus and it shows discrepancies between individual genes and the underlying species tree [8,34,35]. Alternatively, the complete analyses of mitochondrial genomes offer a wealth of high-resolution input and can resolve problems related to taxonomic conflicts and the history of such *D*. (*Ctenodaphnia)* species [36]. Additionally, a combination between traditional morphological taxonomy with molecular and genetic tools are essential for better phylogeny of faunistic studies [37].

Even among the water fleas—being a very old group [38]—*D. arabica* represents a relict lineage, differentiated much earlier from the Gondwanan ancestor [29], so we were not surprised to find its divergence from other daphniids, even at the genomic level.

At the same time, the demographic analysis with a bottleneck effect is consistent with a very strong reduction in the genetic diversity in the species compared to other *Daphnia* species that had already occurred in the Pleistocene. Interestingly, the subsequent expansion happened around 100 Ka, during Marine Isotope Stage 5 (MIS5), with a great fluctuation in the humidity in the Arabian Peninsula including extra-dry episodes [39,40]. Most probably at this time, other species of *Daphnia* in the Arabian Peninsula had passed through a mass extinction due to unstable conditions including the periods of extremely high temperatures and extremely low humidity. Such extinction occurred in the Late Pleistocene in different regions of Eurasia [41,42] and North Africa [43]. These times are also very important as “opportunities for modern human dispersal” through the Arabian Peninsula [40].

Yampolsky et al. [44] studied the functional genomics of acclimation and adaptation in response to thermal stress in *Daphnia pulex* and concluded that “a large number of genes responded to temperature, and many demonstrated a significant genotype-by-environment (GxE) interaction”. Here, in the genome of *D. arabica*, we found some traces of a special adaptation to desert conditions. Specifically, its genome contains an important domain, the C subunit of SOSS (sensor of ssDNA), which was missing in all of the other studied taxa of *Daphnia*. This complex, which consists of a few subunits (A, B and C), contributed to the maintenance of the genome stability (i.e., DNA reparation under stress that creates its breakage) [45,46,47]. Since it is present in *D. arabica* and given the environmental stress that the isolated species faced (mainly the high temperatures), we propose that the SOSS-C subunit could play a role in maintaining the fitness and survival of this species to adapt to the desert environment [45]. The SOSS-C was previously recorded in different animals [48]. There are many sequences in the GenBank to date, but the SOSS-C subunit function has never been discussed in the context of desert animals. 

The absence of a critical subunit of a multicomponent protein complex often destabilizes the complex [49], but we need to conclude that missing the SOSS-C in most *Daphnia* taxa was not critical for them. In contrast, this genus came to be an example of a greatly successful animal in continental waters. Moreover, bearing in mind that the separation of subgenera took place before the *D. arabica* differentiation, we need to hypothesize that SOSS-C was independently lost in different lineages of *Daphnia*, as its secondary “appearance” in a single taxon seems to be a less realistic scenario. Unfortunately, no information of the SOSS-complex in other cladocerans and branchiopod crustaceans is available to date.

In conclusion, the sequenced genome of the newly discovered *Daphnia* will pave the way for future research to identify positively selected genes that are gained or lost in the species and are able to underpin key genes involved in the adaptation of the species to this harsh environment. In addition, our findings will assist in the generation of the crisps of freshwater water fleas, to which we have added this gene, that will be able to tolerate higher global temperatures that are an imminent threat to different ecosystems including diverse freshwater bodies. We believe that it is possible to generate a modified freshwater *Daphnia* using the *D. arabica* SOSS subunit C and subject the modified species to a range of temperatures, followed by viability and genome stability measures.

## 4. Materials and Methods

### 4.1. D. arabica Isolation

Parthenogenetic females of *D. arabica* were hatched from the ephippia (modified molting exuvia containing resting eggs) found in the sediment core collected from its type locality: a dry basin behind Al Shuwaib Dam, which is located near Al Ain City, Abu Dhabi, UAE (24°46′18.8″ N and 55°48′15.2″ E) [26]. The core sediments were poured into a 2 L beaker and rinsed with desalinated bottled commercial drinking water at room temperature (20 ± 1 °C), under a 12:12 h light/dark condition for about 2 weeks. In the third week, a few drops of freshly harvested unicellular monoclonal culture of *Chlorella* sp. were added to the surface water that covered the sediments. A few days later, juveniles were observed on the sediment–water interface. These were transferred to a Petri dish of clear drinking water. The moving juveniles were picked out using a plastic dropper and placed in a 500 mL beaker that contained desalinated commercial drinking water. They were fed every other day at the above-mentioned laboratory conditions.

Under a stereomicroscope, single parthenogenetic females of *D. arabica* were isolated and reared in a 250 mL glass beaker under laboratory conditions. Newborns were isolated in a larger (500 mL) beaker and left to grow. The third generation produced from the grown adults were then reared in a 2-L beaker and reared under lab conditions, until maturation. For molecular analyses, >60 mature females were isolated and preserved in ethyl alcohol (96%) in an Eppendorf cuvette.

Prior to the species being formally identified, its parthenogenetic female, and gamogenetic females and males (Figure 1A) were described morphologically. A few Sanger sequences were deposited in GenBank, and a preliminary phylogenetic analysis was made based on the mitochondrial 12S, 16S, and COI fragments [29]. 

### 4.2. Genomic DNA Isolation and QC

From the 96% ethanol fixed *Daphnia* sample, high-quality genomic DNA was isolated using a QIAamp DNA Mini Kit (Qiagen, Valencia, California, USA; Cat no. 51306) using the tissue protocol. Isolated genomic DNA quality was confirmed using agarose gel electrophoresis and quantitated on a NanoDrop 2000 spectrophotometer (ThermoFisher Scientific™, Waltham, MA, USA) and Qubit Fluorometer (QubitdsDNA HS Assay Kits, Cat no. Q32851; ThermoFisher Scientific™, Waltham, MA, USA). 

### 4.3. Whole Genome Sequencing Library Preparation

For this study, we generated high-depth Illumina shotgun data and Nanopore (MinIon) based long read data. Illumina compatible whole genome shotgun library for the *Daphnia* sample was prepared using the NEBNext® Ultra™ II DNA Library Preparation Kit and sequenced using Illumina NovaSeq 6000 (150 bp paired end (PE) sequencing chemistry). Long read whole genome sequencing (WGS) was carried out using the Oxford Nanopore platform. Oxford Nanopore WGS libraries were prepared using the ligation sequencing kit (SQK-LSK 109; Oxford Nanopore, Oxford, UK) and WGS sequencing was performed on an Oxford Nanopore MinION system (flow cell, FLO-MIN106D R9.4 revision D chip; Oxford Nanopore).

### 4.4. Transcriptome Sequencing

From the sample containing parthenogenetic females of *D. arabica*, the total RNA was isolated using Maxwell (R) RSC simply RNA Tissue Kit. The quality and quantity of isolated RNA were confirmed by agarose gel, NanoDrop2000, and Qubit. The RNA-Seq library was prepared using the directional lib (Ribo-Zero™ rRNA Removal Kits and NEB Next Ultra^TM^ Directional RNA Library PrepKit, New England Biolabs, MA, USA) kit and sequenced in an Illumina NovaSeq machine. The generated transcriptome was used for the downstream gene prediction process. 

### 4.5. Sequencing Data Quality Check and Trimming

The raw Illumina data (both WGS and transcriptome) quality were confirmed using the FastQC tool [50] and the low-quality, adapter, and N-regions present in the reads were trimmed using Trimmomatic v.0.39 software [51]. The sequencing errors found in the Nanopore-MinION reads were corrected and trimmed using CANU v.1.8 [52] software. 

### 4.6. Genome Size Estimation Using Shot Gun Data

We estimated the theoretical genome size of the isolated *D. arabica* using Illumina shot-gun data by the k-mer based approach. All the k-mers (21-mer) present in Illumina PE reads were mined and a k-mer based histogram file was generated using Jellyfish v.2.3.0 software [53]. The theoretical haploid genome size of the *D. arabica* was estimated from the k-mer histogram file using thee GenomeScope v.1 tool [54]. 

### 4.7. Genome Assembly and QC

We carried out hybrid de novo genome assembly of *D. arabica* using both shot-gun and long reads in MaSuRCA v.4.0.4 software [55] for whole-genome assembly that included both the Illumina and Nanopore trimmed reads. The sequencing read error found in the assembled genome was corrected using the Pilon v.1.23 program [56]. From the final genome assembly, the genome size, number of contigs, N50 value, and GC content were calculated and the genome assembly completeness was confirmed by the BUSCO v.4.1.4 tool (using arthropoda_odb10 db) [57]. Furthermore, the genome assembly quality was confirmed by aligning the Illumina WGS reads against the assembled genome. Similarly, the transcriptome reads generated for this study were aligned against the assembled genome and confirmed the assembled genome quality based on the read alignment percentage.

### 4.8. Mitogenome Annotation and Phylogenetic Tree Construction

From the final whole genome assembly, we separated the mitochondrial genome. Mitogenome annotation (CDS, rRNA, and tRNA annotation) was performed using the MITOS tool [58] and the mitogenome map was generated using the Proksee tool (https://proksee.ca/, accessed on 1 December 2022). For phylogenetic tree construction, 22 already published mitogenomes of *Daphnia* (Table S8) were retrieved from the NCBI database and the coding regions were annotated using the MITOS tool. Furthermore, all coding regions were aligned using the MUSCLE program [59] and a coding region based phylogenetic tree was constructed by the MEGA v.X tool [60] using the ML method (bootstrap value 1000).

### 4.9. Gene Prediction and Annotation

After genome assembly, we masked the repeat regions found in the *Daphnia* genome using RepeatsModular v.2.0.1 [61] and the RepeatMasker v.4.1 tool [62]. For the genome annotation, we used both homology-based and ab initio-based gene prediction methods. Generated transcript reads were aligned against the assembled genome using the HiSat v.2.1.0 tool [63] and possible expressed portions (exons or transcripts) of the genome were assembled using StringTie v.2.1.3 tools [64]. These identified transcripts were used as evidence for the gene prediction. Additionally, we retrieved proteins from closely related species and used them for the homology-based gene prediction. Initial gene prediction was carried using the BRAKER v.2.1.5 [65] pipeline (using Augustus v.3.3.3 [66], GeneMark v.4.61 [67], and EVM v.1.1.1 [68] and the final gene prediction was obtained using the MAKER v.3.01 pipeline using Augustus, GeneMark, EVM, and SNAP [69]. Both tRNA and rRNA genes found in the genome were predicted using tRNscan-SE v.2.0.6 [70] and RNAmmer v.1.2 [71]. Predicted proteins were similarity searched against the NCBI-NR and Uniprot-trEMBL Protein Database using thee BlastP program (e-value: 0.000001) [72]. Furthermore, the predicted proteins were functionally annotated using InterProScan v.5.51.85 [73]. Metabolic pathway genes were annotated from the predicted genes using KEGG-KAAS [74], while for pathway analysis, *Daphnia pulex* and *Penaeus vannamei* were considered as reference organisms.

### 4.10. Diversity and Comparative Genomic Analysis

We used our InterProScan results for our isolated *D. arabica* and compared it with the available annotated genome of *Daphnia* from the NCBI (*D. pulex, D. magna, D. galatea,* and *D. pulicaria*), and identified shared PFAM domains among the different species as well as the unique PFAM for each species. We estimated the nucleotide diversity of ANGSD [75] for each of the species using Illumina shot gun reads.

### 4.11. Evolutionary and Demographic History

We applied an assembly and alignment-free (AAF) method (https://sourceforge.net/projects/aaf-phylogeny, accessed on 7 November 2022) [76] using K = 25 to construct the phylogeny of unassembled genomic sequences of *Daphnia* species available on the NCBI short archive (SRA). The divergence time estimate was carried out by running the tool r8s [77] to convert the newick tree generated using the AAF method [76] into the ultrametric tree, where we used a known calibrated adjusted divergence time from TimeTree (http://www.timetree.org, accessed on 10 November 2022) between *Daphnia pulex* and *Daphnia magna* and found it to be 131 Mya. Note that this estimation is somewhat younger compared to a widely used 145 Mya by Kotov and Taylor [78], but also could be applied to such analysis. The whole genome synteny plots between *Daphnia arabica* and the available genomes of *D. sinensis* and *D. pulex* were generated using D-genie [79]. Effective population size history was estimated using the pairwise sequentially Markovian coalescent (PSMC) model, following the pipeline by Li et al. [80]. BAM alignments from *D. arabica* were used to create a consensus sequence using samtools, vcfutils, and bcftools [80]. We performed PSMC analysis using the default parameters recommended by the authors of this method, and we chose an average mutation rate of 8.9 10−9 as well as the generation time of 1 year following Eddie et al. [81].

## Figures and Tables

**Table 1:** *D. arabica* whole genome assembly statistics.

	*D. arabica* Genome	*D. arabica* Mitogenome
Total sequences	453	1
Genome size	116,021,024	16,588
A + T%	~59.2	~69.7
G + C%	~40.7	~30.2
*n* %	0.0001	0
Minimum sequence length	1179	16,588
Maximum sequence length	4,005,661	16,588
N50 length (bp)	1,139,068	16,588
L50 number	30	1
Length 1001–3000 bp	9	0
Length 3001–5000 bp	10	0
Length 5001–7000 bp	7	0
Length 7001–10,000 bp	0	0
Length 10,001–0.1 Mb bp	249	1
Length 100,001–1 Mb bp	134	0
Length > 1 Mb bp	35	0
Protein coding genes	24,041	13
tRNA genes	5374	23
rRNA genes	643	2

## Data Availability

The sequencing data generated during this study were submitted to the NCBI-SRA database under the project id: PRJNA904511. The assembled genome of *D. arabica* along with the annotation was deposited at the Zenodo repository. URL: https://doi.org/10.5281/zenodo.7408613.

## Supplementary Materials

The following supporting information can be downloaded at: https://www.mdpi.com/article/10.3390/ijms24010889/s1.
