# Supplementary figures and images for: Genome Assembly of a Relict Arabian Species of Daphnia O. F. Müller (Crustacea: Cladocera) Adapted to the Desert Life
Source: Int J Mol Sci. 2023 Jan 3;24(1):889. doi: 10.3390/ijms24010889 (PMC9820869; doi:10.3390/ijms24010889)

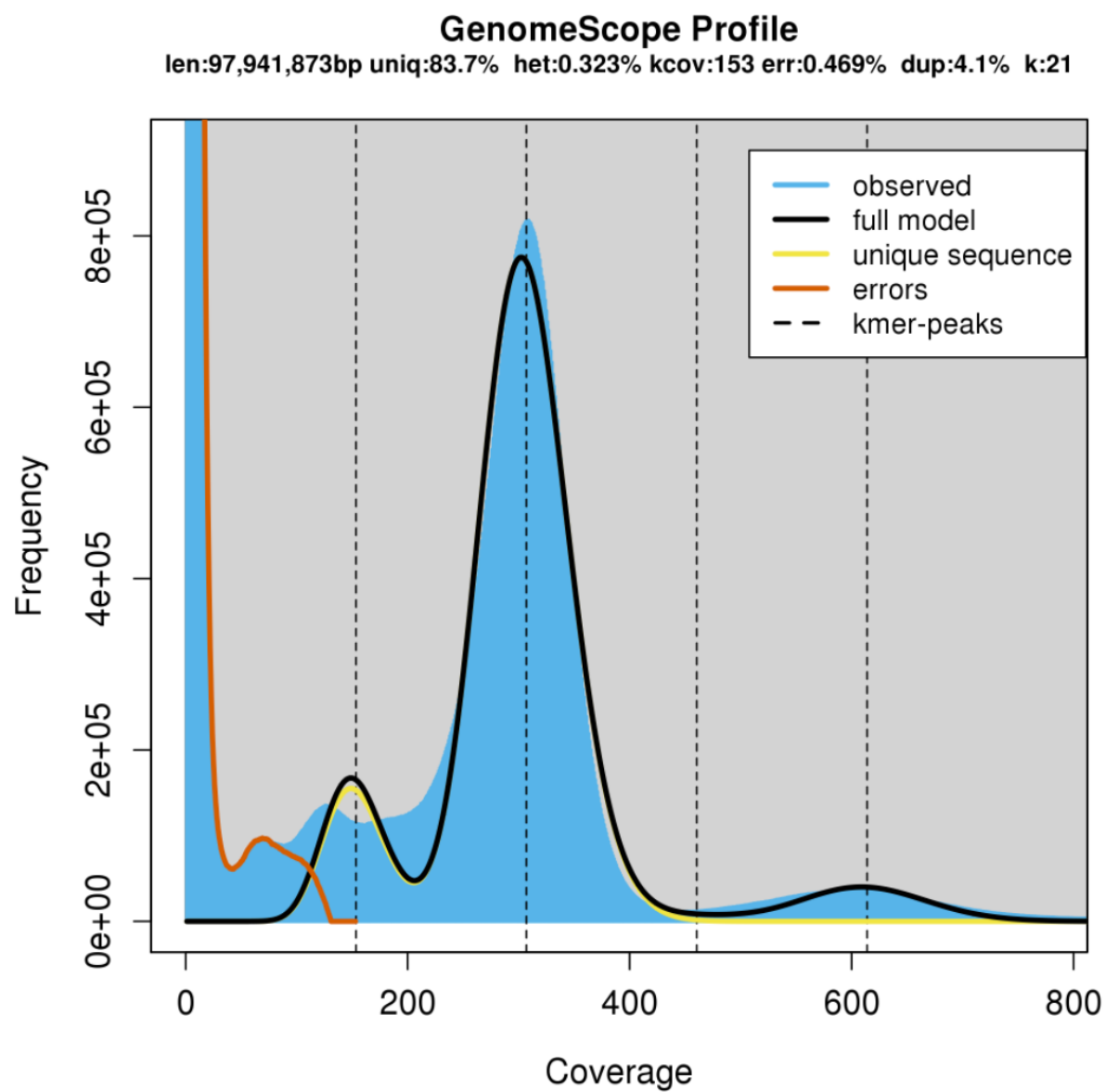

Figure S1. *D. arabica* k-mer (21-mer) based genome size estimation

Supplement: Supplementary file 1 [file ijms-24-00889-s001.zip › sup_figures/Figure_S1.pdf]
